# Anemia management after acute brain injury

**DOI:** 10.1186/s13054-016-1321-6

**Published:** 2016-06-17

**Authors:** Christophe Lelubre, Pierre Bouzat, Ilaria Alice Crippa, Fabio Silvio Taccone

**Affiliations:** Department of Intensive Care, Hôpital Erasme, Université Libre de Bruxelles (ULB), Route de Lennik 808, 1070 Brussels, Belgium; Laboratoire de Médecine Expérimentale, Université Libre de Bruxelles (ULB) - Unité 222, CHU Charleroi (Hôpital André Vésale), Rue de Gozée 706, Montigny-Le-Tilleul, Belgium; Department of Anaesthesiology and Critical Care, Grenoble University Hospital, Hôpital Albert Michallon, Avenue Maquis du Grésivaudan, Grenoble, F-38043 France; Grenoble Neurosciences Institute, Grenoble Alpes University, Grenoble, F-38043 France

**Keywords:** Red blood cell transfusion, Threshold, Anemia, Traumatic brain injury, Subarachnoid hemorrhage

## Abstract

Anemia is frequent among brain-injured patients, where it has been associated with an increased risk of poor outcome. The pathophysiology of anemia in this patient population remains multifactorial; moreover, whether anemia merely reflects a higher severity of the underlying disease or is a significant determinant of the neurological recovery of such patients remains unclear. Interestingly, the effects of red blood cell transfusions (RBCT) in moderately anemic patients remain controversial; although hemoglobin levels are increased, different studies observed only a modest and inconsistent improvement in cerebral oxygenation after RBCT and raised serious concerns about the risk of increased complications. Thus, considering this "blood transfusion anemia paradox", the optimal hemoglobin level to trigger RBCT in brain-injured patients has not been defined yet; also, there is insufficient evidence to provide strong recommendations regarding which hemoglobin level to target and which associated transfusion strategy (restrictive versus liberal) to select in this patient population. We summarize in this review article the more relevant studies evaluating the effects of anemia and RBCT in patients with an acute neurological condition; also, we propose some potential strategies to optimize transfusion management in such patients.

## Background

Anemia is a frequent condition among critically ill patients [1] and appears early during their hospital course. In a European, multicenter study on 3534 patients, 63 % of those newly admitted to the intensive care unit (ICU) had hemoglobin (Hb) levels below 12 g/dl on admission; in particular, 29 % of them had Hb values below 10 g/dl [2]. Overall, 37 % of these 3534 patients received at least one red blood cell transfusion (RBCT) during their ICU stay. Interestingly, during the first 28 days, Hb levels tended to reach 10 g/dl on average regardless of the initial Hb value or the occurrence of bleeding events. In another study on 4892 ICU patients in which 44 % received at least one RBCT after a median of 3 days, an Hb level below 9 g/dl was associated with poorer outcomes whereas the amount of RBCT transfused was also associated with increased ICU length of stay and mortality [3].

The pathophysiology of anemia in critically ill patients remains multifactorial and has been compared to the so-called “anemia of chronic illness”; as such, apart from evident causes such as primary blood losses (e.g., trauma, surgery, gastrointestinal bleeding), multiple other etiologies contribute and often coexist in the same patient [4]. These include, amongst others, blood losses related to minor procedures or phlebotomy, hemodilution secondary to fluid resuscitation, altered red blood cell (RBC) production, and reduced RBC half-life [5–7].

Although it is associated with poor outcome, whether anemia exerts deleterious consequences on brain function in various pathological conditions remains a matter of debate. In neurological circumstances such as severe traumatic brain injury (TBI) or stroke, Hb level is a primary determinant of brain oxygenation and, in a recent study, anemia was an independent predictor of mortality among patients suffering from an acute ischemic stroke [8, 9].

In this article, we summarize the literature evaluating the effects of anemia and RBCT in patients with an acute neurological condition as well as potential strategies to optimize Hb management in such patients.

### Effects of anemia on the brain

Oxygen delivery (DO_2_) to the brain is directly proportional to cerebral blood flow (CBF) and arterial oxygen content (CaO_2_) and, therefore, also to Hb levels according to the equation:

$$ \mathrm{D}{\mathrm{O}}_2 = \mathrm{Q} \times \mathrm{C}\mathrm{a}{\mathrm{O}}_2 $$

where Q indicates blood flow and CaO_2_ = Hb × SaO_2_ × 1.39 (SaO_2_ indicates arterial oxygen saturation). According to this equation, a significant reduction of Hb may lead to decreased brain DO_2_ and eventually tissue hypoxia if the compensatory mechanisms aiming to keep a constant tissue oxygenation fail or are overtaken [10]. In the setting of normovolemic anemia, these mechanisms include the activation of carotid and aortic chemoreceptors and, hence, of the sympathetic tone, which lead to a rise in heart rate and left ventricular stroke volume, resulting in increased cardiac output and CBF [11]. Oxygen extraction is also increased at the microcirculatory level [12]. Moreover, anemia is associated with reduced blood viscosity, which promotes venous return and decreases the resistance to systemic flow as well as reduces endothelial shear stress, resulting in an improved microvascular perfusion [13, 14].

In this setting, cerebral vasodilation is promoted by enhanced production of nitric oxide (NO) by perivascular neurons and endothelial cells [15]. This results in an increased CBF that maintains a constant cerebral DO_2_. Importantly, according to Poiseuille's equation showing that blood flow is proportional to the vessel radius to the fourth power, small changes in vascular diameter will have a large influence on CBF. The role of NO in regulating CBF during anemia is extremely complex: cerebral hypoxic vasodilatation can be altered by NO synthase (NOS) inhibitors through a direct effect on neuronal NOS (nNOS) [15], while expression of endothelial NO synthase (eNOS) is increased by increased capillary shear stress [16]. Finally, oxyhemoglobin is also able to transport NO; following oxygen release from oxyhemoglobin, structural changes in heme conformation could promote NO release and further promote local vasodilation in territories with high oxygen extraction [10]. Interestingly, cerebral hypoxia secondary to severe anemia would also induce the production of the transcription factor hypoxia-inducible factor (HIF), an heterodimer composed of two subunits (HIF-1α and HIF-1β) which plays an important role in the protection of brain cells from ischemia [17]. Moreover, HIF also promotes the secretion of erythropoietin (EPO), which negatively regulates neuronal apoptosis and seems to exert some neuroprotective effects through specific receptors expressed within cerebral tissue [18], and of vascular endothelial growth factor, which stimulates angiogenesis and allows long-term adaptations to tissue hypoxia [19].

**Fig. 1:**
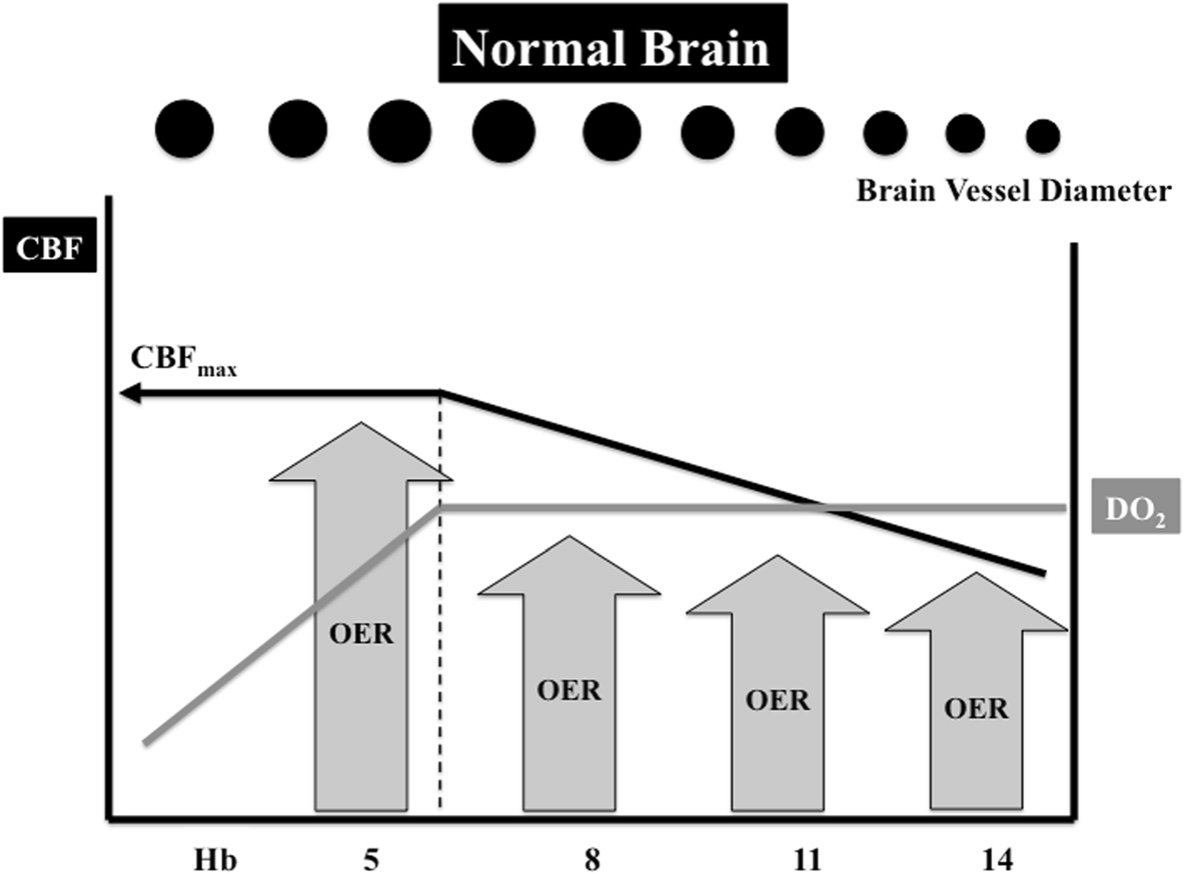
In the normal brain, a progressive reduction of hemoglobin (*Hb*) is compensated for by vasodilation, which results in increased cerebral blood flow (*CBF*, *black line*) and a constant cerebral oxygen delivery (*DO*_*2*_, *grey line*). When Hb falls below 5–6 g/dl, DO_2_ is progressively reduced; no further vasodilation can occur and maximal CBF values (*CBF*_*max*_) are obtained. The oxygen extraction rate (*OER*) then increases to meet metabolic tissue requirements

**Fig. 2:**
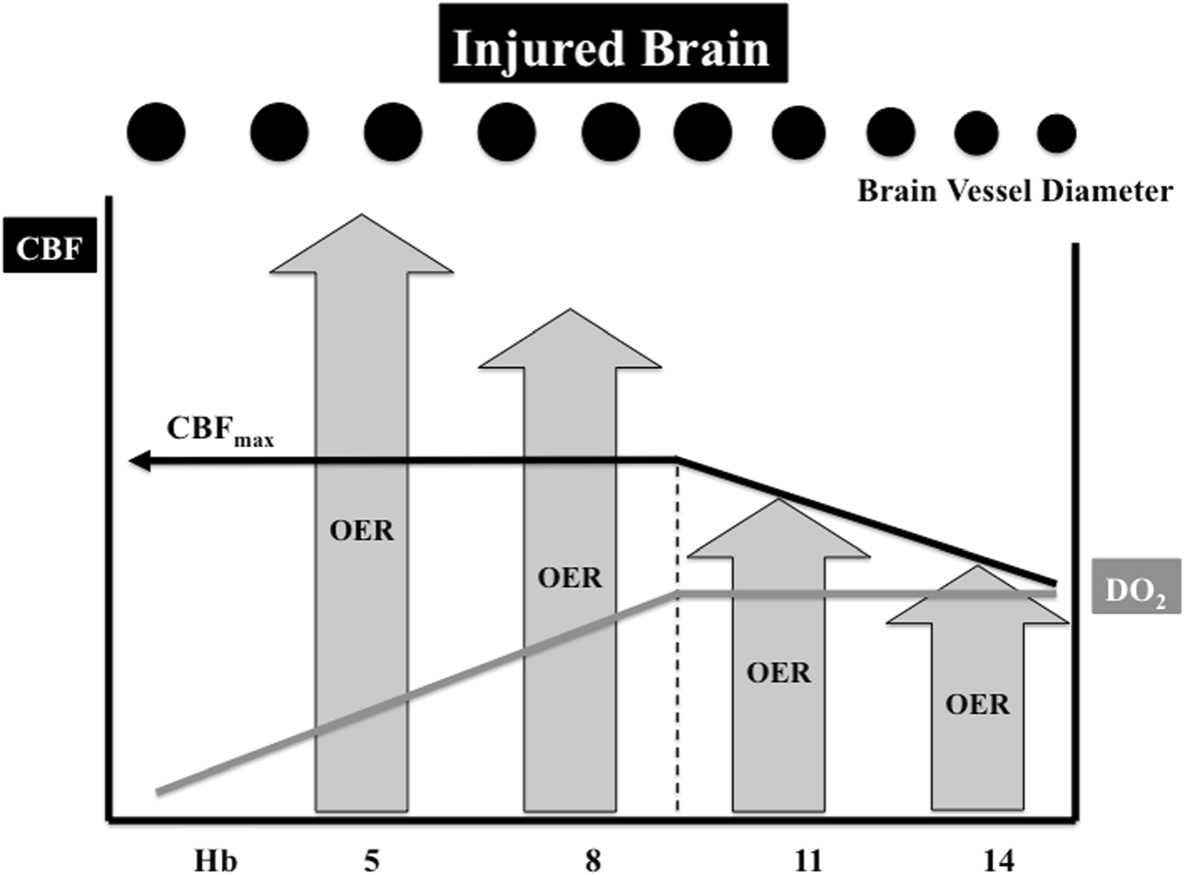
In the injured brain, cerebral blood flow (*CBF*, *black line*) is lower than in the normal brain at the corresponding Hb levels. As the “cerebrovascular reserve” is compromised in this setting, the maximal CBF (*CBF*_*max*_) will be possibly obtained at Hb levels around 8–9 g/dl and any further decrease of Hb below these thresholds will contribute to reduce cerebral oxygen delivery (*DO*_*2*_, *grey line*). The oxygen extraction rate (*OER*) then increases to meet metabolic tissue requirements above these thresholds

Overall, these adaptation mechanisms maintain DO_2_ during anemia in healthy conditions, at least until a critical Hb threshold below which tissue hypoxia and altered brain function may develop. As such, in healthy volunteers submitted to progressive normovolemic anemia, some authors observed increasing fatigue and cognitive disorders (short- and long-term memory disturbances) when Hb levels fell to 5 g/dl; these symptoms were rapidly reversed after autologous RBCT [14, 20]. In healthy subjects, however, acute normovolemic anemia (to Hb levels of 5.1 ± 0.3 g/dl) was not associated with increased somatosensory-evoked potential latencies, suggesting brain dysfunction [21]. These findings underline that a progressive reduction of Hb in the normal brain can be compensated by an increase in CBF due to cerebral vasodilation until a critical Hb level around 5–6 g/dl, when cerebral DO_2_ will be progressively reduced as no further vasodilation can occur and maximal CBF values are obtained (Fig. 1). Nevertheless, these data on healthy volunteers may not directly translate to brain-injured patients. First, Hb levels in these critically ill patients are generally higher than those used in the aforementioned studies [22]. Second, some of these brain-injured patients may develop hemodynamic instability or acute heart failure, which would significantly impair the compensatory increase in cardiac output to provide adequate cerebral oxygenation during anemia [23]. Third, anemia-induced vasodilation may be limited by other ongoing compensatory mechanisms to maintain adequate brain perfusion induced by the acute brain injury itself, so that the “cerebrovascular reserve”, i.e., the capability of brain vasculature to vasodilate in response to different stimuli (including changes in mean arterial pressure, arterial carbon dioxide tension (PaCO_2_), or reduced DO_2_), is significantly limited when compared with healthy subjects [24]. This could promote brain tissue hypoxia at hemoglobin levels higher than the hemoglobin thresholds observed in healthy volunteers [25]. Finally, brain lesions observed after TBI or a stroke may be highly heterogeneous; some territories defined as "penumbra" zones (e.g., moderately ischemic tissue lying between tissue that is normally perfused and an infarcted area) exist, where oxygen supply may become inadequate to satisfy oxygen demand in the case of anemia. Thus, in the injured brain, a lower CBF compared with the normal brain is observed for similar Hb values (Fig. 2). As the “cerebrovascular reserve” is compromised in this setting, maximal vasodilation may occur at Hb levels around 8–9 g/dl and any further decrease in Hb below this threshold may contribute to reduced cerebral DO_2_.

### Effects of anemia on the injured brain

#### Traumatic brain injury

Several studies have shown an association between anemia and poor outcome after TBI [26–28]. In two post hoc analyses including more than 3500 patients with severe TBI (defined as a Glasgow Coma Scale score <9), low Hb levels were significantly associated in a logistic regression model with poor neurological outcome at 3 and 6 months after the initial insult [28, 29]. In a retrospective study on 1150 patients with TBI, anemia (defined as an Hb level below 9 g/dl) was significantly associated with increased mortality (adjusted odds ratio (OR) 3.67, 95 % confidence interval (CI) 1.13–2.24) [26]. In another retrospective study on 169 patients with TBI, an average Hb level below 9 g/dl over a period of 7 days was associated, in a multivariate analysis, with increased hospital mortality (relative risk 3.1, 95 % CI 1.5–6.3; *p* = 0.03) [27]. Similar observations were found in two other studies [30, 31], although the evaluation of anemia and poor outcome after TBI did not consistently demonstrate harm in others [32–34]. Unfortunately, these studies generally had important methodological weaknesses. Most of them were retrospective, which may have reduced the collection of variables potentially influencing outcome. Also, anemia was defined according to different cutoffs, thus limiting comparison among all the different cohorts of patients. Also, only one single Hb measurement was considered in the definition of anemia, while the exposure of an injured brain to prolonged periods of low Hb levels may be more relevant. As an example, in 116 patients with severe TBI, Griesdale et al. [35] observed that a Hb time curve above 9 g/dl was associated with improved neurological outcome independent of the administration of RBCT. Finally, the primary outcome evaluating the relationship between anemia and outcome was mortality in some studies while others focused on long-term neurological recovery [36].

Furthermore, anemia has also been associated with changes in brain metabolism or oxygenation. As such, Sahuquillo et al. [37] showed that, in 28 patients with severe TBI, low Hb levels were one of the most important predictors of the development of ischemic areas, as suggested by increased arterio-jugular oxygen (AJDO_2_) and lactate differences. Similarly, Cruz et al. [38] evaluated the cerebral metabolic rate of oxygen consumption (CMRO_2_) in TBI patients with anemia and found that a decrease of Hb was associated with a decrease in CMRO_2_ independent of the level of consciousness of patients. However, anemia (defined as Hb <11 g/dl in at least three measurements) was associated with a marked decrease in AJDO_2_ in only 9 % of the observations in another study [39], suggesting that global cerebral ischemia is a rare finding in acute brain injury with anemia. More recently, in a retrospective analysis of 474 simultaneous measures of Hb and brain oxygen tension (PbtO_2_) in 80 TBI patients, only Hb levels <9 g/dl were significantly associated with a low value of PbtO_2_ (<20 mmHg). In this study, anemia combined with low PbtO_2_ was associated with poor neurological outcome (Glasgow Outcome Scale of 1–3 at 30 days), whereas isolated anemia was not [40]. Association between anemia and low PbtO_2_ was, however, not found in all studies [41].

#### Subarachnoid hemorrhage

In several studies including patients suffering from subarachnoid hemorrhage (SAH), anemia was an independent risk factor for poor neurological outcome [42, 43]. In a retrospective study on 580 patients with SAH, anemia was an independent risk factor for mortality and neurological disability at 3 months, even after correction for confounders (OR 1.8, 95 % CI 1.1–2.9, *p* = 0.02) [44]. In another retrospective study (*n* = 245), Hb below 10 g/dl was associated with poorer outcomes, including mortality, severe disability, and the development of delayed cerebral ischemia [45]. Conversely, in a large cohort of SAH patients (*n* = 611), higher Hb levels were found in patients with good outcome compared with those with poor outcome (11.7 ± 1.5 versus 10.9 ± 1.2 g/dl, *p* < 0.001) [46]; also, the highest Hb values over the ICU stay were an independent predictor of good neurological recovery at 3 months.

As for studies on TBI, the effects of anemia on brain metabolism have also been evaluated in SAH patients. In a prospective study on 20 patients with poor-grade SAH, Hb levels below 9 g/dl were associated with an increased risk of low PbtO_2_ values (<20 mmHg) and altered metabolism (e.g., increased lactate to pyruvate ratio (LPR) above 40 when assessed using cerebral microdialysis catheters), suggesting ongoing anaerobiosis in the absence of adequate cerebral DO_2_ [47]. Also, in a retrospective analysis of 359 different measurements performed in 34 SAH patients, Hb levels below 9–10 g/dl were independent predictors of tissue hypoxia (e.g., elevated LPR) compared with higher Hb levels [48].

#### Other forms of brain injury

Anemia on admission has been found to be among the most significant predictors of short- and long-term poor outcome in patients with acute ischemic stroke [49]. In young patients suffering from acute stroke due to cervical artery dissection (*n* = 1206), anemia (defined as Hb <12 g/dl) was found in 7 % of them on admission and was associated with the severity of stroke and unfavorable neurological outcome [50]. In another study, anemia on admission (identified as an hematocrit value less than 30 %) was associated with poor outcome in patients with less severe stroke, defined as a National Institutes of Health Stroke Scale score of <10 [51]. Nevertheless, mild anemia could worsen patients’ functional status also when occurring in the sub-acute phase of stroke [52]. Also, decreasing Hb levels after admission could independently predict infarct growth in stroke patients treated with intravenous thrombolysis [53]. In contrast, in a recent retrospective study, Hb concentrations higher than the normal limits on admission were also associated with greater disability at discharge and 30-day mortality, even after adjustment for major potential confounders, after ischemic stroke [54].

In one retrospective study, anemia on admission was identified in 19 % of patients with a non-traumatic intracranial hemorrhage (ICH) and was found to be an independent predictor of long-term mortality [55]. Also, lower Hb levels (<12 g/dl) were found in 23 % of 2406 ICH patients during their hospital stay, including 4 % with Hb <10 g/dl [56]. Patients with anemia were more likely to have severe neurological deficits on admission, in particular when ICH was not associated with the use of anticoagulants. Hb below 10 g/dl was also associated with poor outcome and increased 1-year mortality. Similar results were also found in other studies [57, 58]. Interestingly, anemia was also a predictor of larger hematoma volumes in these patients [59].

Among patients suffering from post-anoxic brain injury, Ameloot et al. [60] found a strong linear relationship between Hb and cerebral brain oxygen saturation (StO_2_), assessed by non-invasive near-infrared spectroscopy. Moreover, Hb levels below 10 g/dl generally resulted in low StO_2_ values, while Hb values above 12.3 g/dl were associated with better outcome, particularly in patients with StO_2_ values <62 %.

### Efficacy of RBCT in patients with acute brain injury

Considering the frequent association of anemia and poor outcome after an acute brain injury, current recommendations on the use of a restrictive transfusion strategy in ICU patients (transfusion if Hb levels <7 g/dl in the absence of severe cardiac comorbidities) [61] may not apply to patients with brain injuries. One may argue that RBCT to increase Hb levels above 9–10 g/dl in these patients would be a logical therapeutic decision to reduce the risk of tissue hypoxia and potentially improve patient outcome. Nevertheless, the benefits of RBCT should always be weighed against the risk of a transfusion-related complication; as such, RBCT was associated with increased mortality and a higher occurrence of organ dysfunction among critically ill patients, although this has been observed only in observational studies [62]. The pathophysiology of such complications is complex and is related to several pathways, including immune modulation [63], risk of circulatory overload [64] or acute lung injury [65], altered RBC function due to prolonged storage [66], or impaired peripheral microcirculation due to NO scavenging and reduced deformability [67].

#### Severe traumatic brain injury

In some recent studies on patients with severe TBI, the increase in PbtO_2_ after RBCT was generally small [68–71]; moreover, RBCT was associated with a decreased PbtO_2_ in some subjects. These studies included no clear prediction criteria to distinguish between "responders" and "non-responders" (on the basis of PbtO_2_ changes) to RBCT [69, 71]. Even if RBCT could produce an improvement in cerebral oxygenation, this was not always accompanied by significant changes in cerebral metabolism, e.g., reduction in LPR [71].

Alternatively, studies evaluating RBCT as a predictor of good outcome after TBI have found inconsistent results, although some of them suggested detrimental effects [43]. In a large retrospective study on 1150 TBI patients in which 46 % of patients received RBCT when Hb levels were below 9 g/dl, Salim et al. [26] found that RBCT was associated with an increased hospital mortality in a logistic regression model (adjusted OR 2.19, 95 % CI 1.27–3.75, *p* = 0.004), while anemia was not. In another retrospective study on 139 anemic patients (hematocrit between 21 and 30 %) with TBI, RBCT was an independent risk factor for poor neurological outcome at 3 and 6 months [70]. Other studies did not confirm these findings. In a small retrospective study of 82 TBI patients with moderate anemia (Hb between 8 and 10 g/dl), there was no association between RBCT and poor neurological outcome and mortality was similar between anemic patients (Hb between 8 and 10 g/dl) who received RBCT and those who didn’t [68].

#### Subarachnoid hemorrhage

Several studies on patients with SAH found an association between RBCT and worse neurological outcome or even increased mortality [43, 72–74]. In a retrospective study on 245 patients with SAH, RBCT was associated, in a multivariate analysis, with an increased risk of composite endpoint, including mortality, severe disability, or delayed cerebral ischemia (OR 4.3, 95 % CI 1.5–9.3, *p* < 0.01), as well as the occurrence of more nosocomial infections [45]. However, these findings were not confirmed in all studies. In a study on 292 SAH patients, the authors found no association between RBCT and increased mortality or poor neurological outcome in a multivariate analysis [75].

Few studies have evaluated the impact of RBCT on brain oxygenation in poor-grade SAH patients. In one study on 35 neuro-critically ill patients (including 12 with SAH) receiving RBCT (2 units on average; mean Hb threshold for RBCT of 8.7 g/dl), Smith et al. [69] observed only a modest increase in PbtO_2_ (3.2 mmHg) and no relationship between the increase in PbtO_2_ and baseline values of cerebral oxygenation; importantly, in nine patients, PbtO_2_ decreased after RBCT. In another study, Kurtz et al. [76] showed that each 1.0 g/dl increase in Hb levels after RBCT in poor-grade SAH patients was associated with an increase in PbtO_2_ of 1.39 mmHg, without significant effects on cerebral LPR. In a prospective study on eight anemic SAH patients (baseline hemoglobin 8.7 g/dl) in whom a cerebral positron emission tomography (PET) was performed, the administration of 1 unit of RBC resulted in a significant increase in brain DO_2_; these effects were independent of CBF and related to a higher CaO_2_ [77]. In this study, CMRO_2_ remained globally unchanged but there was a significant decrease in oxygen extraction ratio, in particular in cerebral territories with the lowest baseline DO_2_. To confirm that these data were not secondary to the hemodynamic effects (e.g., increased cardiac output or mean arterial pressure through fluid expansion) of RBCT, the same authors evaluated brain DO_2_ in three subgroups of poor-grade SAH patients (nine receiving a fluid bolus, 12 receiving phenylephrine to raise mean arterial pressure without clear vasospasm, 17 receiving one RBCT in case of Hb <11 g/dl—baseline Hb of 9.1 ± 1.2 g/dl) [78]. Regional cerebral DO_2_ significantly increased in the three groups but the effects were more important in patients receiving RBCT; however, the proportion of patients with low DO_2_ was decreased only among patients treated with vasopressors or RBCT, showing the more relevant improvement in global cerebral oxygenation.

#### Other forms of brain injury

Outcomes in ICH patients receiving RBCT have been contradictory. In 546 consecutive patients with ICH, RBCT was administered to 100 patients (18 %) during their hospital stay; in multivariable analysis, RBCT was associated with improved survival at 30 days (OR 2.76, 95 % CI 1.45–5.26, *p* = 0.002) [79]. A recent retrospective study failed to demonstrate an improvement in outcomes with RBCT in patients with ICH [80]. Also, in another study, RBCT was not an independent predictor of improved neurological outcome [81].

Very few data are available on the effects of RBCT on the outcome of patients with ischemic stroke. A recent retrospective study showed that one-third of anemic (Hb <12 g/dl) patients received at least one RBCT at the discretion of the attending physician; although anemia was associated with a longer length of ICU stay and duration of mechanical ventilation requirements, no significant benefit from RBCT was found and no specific transfusion strategies were recommended in this patient population [82].

### Which is the optimal transfusion strategy for patients with acute brain injury?

Only a few studies have compared the effects of two different transfusion strategies on the outcome of patients with an acute brain injury. In a subgroup analysis of the multicenter randomized Transfusion Requirements in Critical Care (TRICC) trial on 67 patients with TBI, patients randomized to the “restrictive” RBCT policy (e.g., Hb of 7.0 g/dl to initiate RBCT, *n* = 29) received less RBC units than those included in the “liberal” strategy (Hb of 10.0 g/dl to initiate RBCT, *n* = 38), with a similar 30-day mortality (17 versus 13 %, *p* = 0.64), hospital length of stay, and development of multiple organ dysfunction [32]. In the subgroup of 66 children with different types of brain injury included in the Transfusion Requirements in the Pediatric Intensive Care Unit (TRIPICU) study, patients were randomized to receive RBCT for an Hb threshold of 7 or 9.5 g/dl [83]; the mortality rate was very low (3/66) and similar between the two groups. In a retrospective study, TBI patients receiving RBCT and who could not reach an Hb target of at least 9.3 g/dl at the end of the initial surgery showed a higher early mortality than others (17/37 (46 %) versus 34/102 (33 %)) [84]. After adjustment for confounders, however, no significant impact of transfusions was found on patient outcome and the difference in mortality was lost after 4 weeks. In two other retrospective studies on TBI [68, 70] including more than 200 patients, patients receiving at least one RBCT were compared with those who were not transfused for a specific range of Hb between 7 and 10 g/dl; no significant effect on outcome was observed.

Only two prospective randomized trials have been conducted specifically in brain-injured patients. In the first study, Naidech et al. [85] randomized 44 SAH patients at high risk of vasospasm to receive RBCT for a target Hb of 10 or 11.5 g/dl; more transfusions were observed in the high Hb threshold group than in the other, while safety endpoints (e.g., infections and thromboembolic events) were not different between the groups. The numbers of cerebral infarctions on cerebral magnetic resonance imaging (MRI; 6/20 versus 9/22) and of patients showing a poor neurological recovery were lower, although not statistically significant, in the higher Hb threshold group. Nevertheless, the primary outcome of this study was the safety of two different RBCT policies and the limited cohort of patients precluded any further analysis of the impact of Hb levels on neurological outcome. In the second study, Robertson et al. [86] investigated the effects of two different thresholds of Hb to guide RBCT (7 versus 10 g/dl) in patients suffering from TBI in a factorial design including also the administration of erythropoietin (EPO) or placebo. On a total of 200 patients, favorable outcome (dichotomized Glasgow Outcome Scale at 6 months) was similar between patients included in the 7 g/dl (37/87, 43 %) or in the 10 g/dl (31/94, 33 %) group , although patients maintained relatively high median Hb levels in both groups throughout the study (between 9.7 and 10.8 g/dl in the “restrictive” transfusion strategy versus 11.0 and 11.5 g/dl in the “liberal” strategy). This study showed that there was no significant benefit in maintaining high Hb levels in patients suffering from severe TBI. Moreover, thromboembolic events were significantly more frequent in the group transfused at 10 g/dl (22/101 (22 %) versus 8/99 (8 %), *p* = 0.009).

### Practices

Two surveys have been published to determine whether physician specialty influences transfusion threshold in patients with TBI or SAH. In the first study, trauma surgeons, neurosurgeons, and ICU physicians from 187 level I trauma centers in the United States were asked to indicate their Hb threshold to initiate RBCT in two clinical scenarios referring to a patient with severe TBI either with or without intracranial hypertension [87]. The response rate was 58 %; neurosurgeons used a greater mean Hb threshold to initiate RBCT than trauma surgeons and ICU physicians whether the intracranial pressure was normal or elevated. Moreover, neurosurgeons used less indicators of poor anemia tolerance, such as increased lactate, low mixed venous saturation, or PbtO_2_, than the others to decide on RBCT. In a second study conducted in North America, neuro-intensivists, vascular neurosurgeons, and multidisciplinary intensivists working in academic hospitals were questioned on the common triggers to initiate RBCT in SAH patients [88]. More than half of the clinicians (282/531) eventually responded. Mean Hb concentrations at which clinicians administered RBCT significantly increased from a good-grade SAH patient to a poor-grade one (7.8 versus 8.2 g/dl), in particular in the case of cerebral vasospasm and delayed cerebral ischemia. Opinions covered a broad range in each setting. Neurosurgeons expressed higher minimum Hb goals than ICU physicians to initiate RBCT. The presence of low PbtO_2_ (<15 mmHg) and high LPR (>40) were also important triggers to administer RBCT.

### A practical approach

Several observational studies have shown that anemia, even if defined with different Hb thresholds, was associated with worse neurological outcome and increased mortality rate after TBI, SAH, and other forms of brain injury. Whether anemia merely reflects a higher severity of the underlying disease, a longer ICU length of stay and other ongoing processes (e.g., active bleeding, sepsis, surgical procedures), or can directly affect the neurological recovery of such patients remains unclear. Interestingly, the effects of transfusions to increase Hb levels in moderately anemic patients remain controversial, with a modest and inconsistent increase in brain oxygenation after RBCT and serious concerns about the risk of increased mortality. Thus, considering this "blood transfusion anemia paradox", the optimal Hb level to trigger RBCT in brain-injured patients has not yet been defined. There is no strong evidence to support targeting a Hb concentration greater than 7 g/dl or a liberal transfusion strategy in this patient population. Importantly, it should be better evaluated whether alternative strategies to RBCT might be considered in this setting to avoid a significant decrease of Hb levels below critical thresholds to ensure adequate brain oxygenation.

**Fig. 3:**
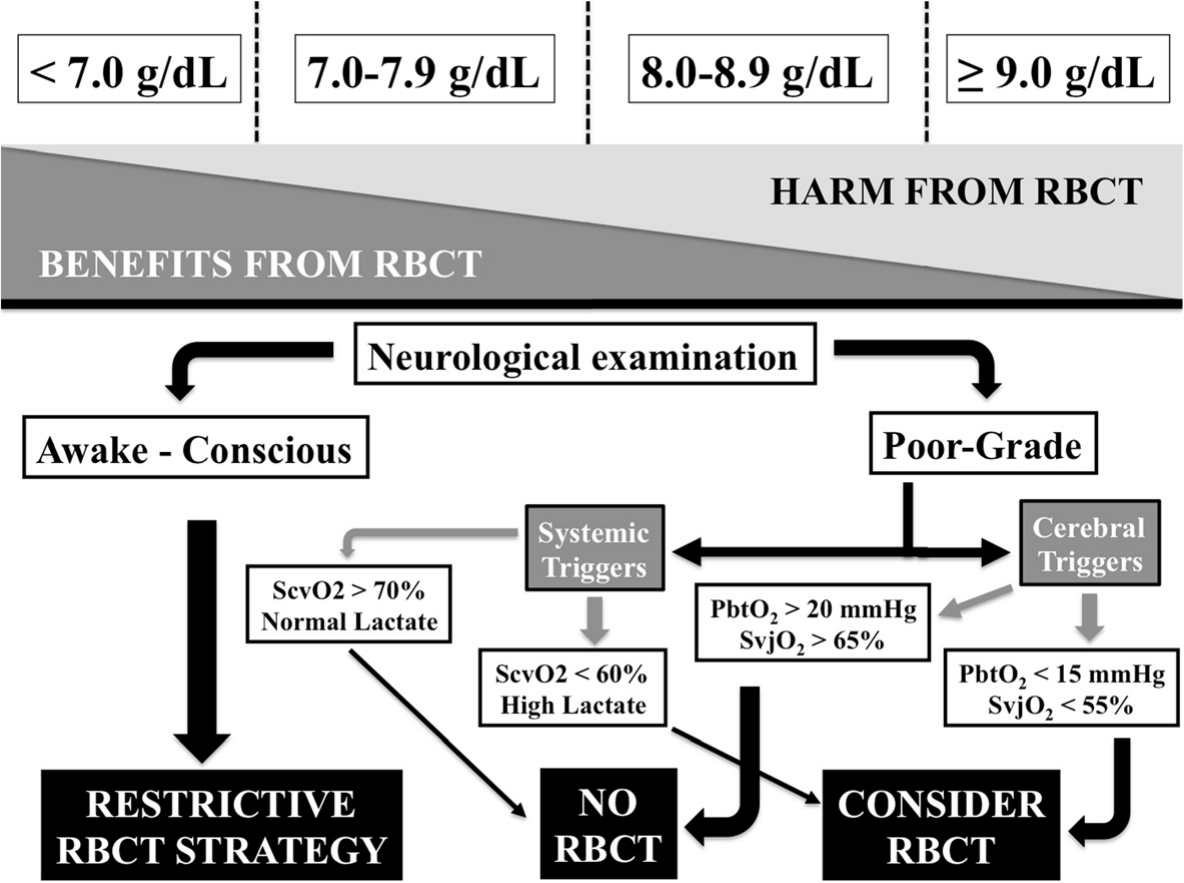
The decision to administer red blood cell transfusions (*RBCT*) should take into consideration the potential benefits and harms of this intervention, according to different haemoglobin (*Hb*) levels at which RBCT is initiated. In brain-injured patients, the RBCT strategy should be “restrictive” (if Hb is less than 7.0 g/dl) in awake and conscious patients. For poor-grade comatose patients, systemic (superior vena cava oxygen saturation (*ScvO*_*2*_) or high lactate levels) or cerebral triggers (jugular vein oxygen saturation (*SvjO*_*2*_) or brain tissue oxygen pressure (*PbtO*_*2*_)) could be used to guide RBC administration

According to what has been proposed for other critically ill patients, a “restrictive” transfusion practice should be considered safe for brain-injured patients who are awake and can undergo repeated clinical examination (Fig. 3). In these patients, RBCT should be administered to maintain Hb levels of at least 7.0 g/dl [89]. In case of neurological deterioration or in poor-grade patients, the decision to initiate RBCT should then be individualized to some specific triggers suggesting a poor tolerance to anemia (e.g., ischemic heart disease) or global/cerebral tissue hypoxia, which may be secondary or at least enhanced by reduced Hb levels. Regarding “systemic” triggers, optimization of oxygen delivery to reach a mixed (SvO_2_) or superior vena cava (ScvO_2_) oxygen saturation >70 % using, amongst all potential interventions, also RBCT, as shown for the early management of sepsis [90], may be useful to improve brain oxygenation and overall outcome. As such, Gaieski et al. [91] showed that early hemodynamic optimization of patients after post-anoxic brain injury using a target ScvO_2_ of ≥65 % was associated with reduced mortality, although not statistically significant, when compared with historical controls (10/20 (50 %) versus14/18 (78 %), *p* = 0.15). Similarly, Walters et al. [92] showed a trend towards better neurological outcome in patients treated with such a therapeutic approach compared with historical controls (31 % versus 12 %, *p* = 0.08). Interestingly, RBC can significantly influence the microcirculation, where their altered morphology and deformability may impair local rheology and further enhance microvascular abnormalities associated with sepsis, particularly in those patients with normal baseline microvascular flow [93]. Similar results were also found for patients with elevated lactate levels [94]. Thus, using biomarkers of impaired oxygen tissue delivery could help to identify those patients who are more likely to benefit from RBCT because of inadequate systemic DO_2_, although no data are available on how these systemic “triggers” can guide RBCT specifically in brain-injured patients.

Specific “cerebral” triggers may be helpful and should include the invasive or non-invasive assessment of cerebral oxygenation (e.g., venous saturation in the jugular vein (SvjO_2_), PbtO_2_, or StO_2_) to individualize transfusion requirements, even though they may suffer technical limitations or poorly predict a “positive” response (e.g., improved oxygenation) to RBCT. Only patients with anemia (e.g., Hb <9–10 g/dl) and concomitant tissue hypoxia (e.g., PbtO_2_ <15–20 mmHg, SvjO_2_ <55 %) should be considered as potential candidates for RBCT. The main limitations of such an approach are that these oxygenation-monitoring devices are not available in all centers and some of them are costly and give information only for a very limited area of the brain [88]; it would thus be difficult to recommend the wide use of such tools in all poor-grade brain-injured patients. Importantly, RBCT is not the only therapeutic intervention that may improve cerebral oxygenation in such patients; as such, clinicians should rule out other possible causes for cerebral hypoperfusion (e.g., increased intracranial hypertension, severe hypocapnia, systemic hypotension) or hypoxia (e.g., seizures, hyperthermia, arterial hypoxemia) before considering RBCT in the management of such patients. Finally, patients may present with signs of low systemic DO_2_ (e.g., low ScvO_2_) and normal cerebral oxygenation (e.g., PbtO_2_ > 20 mmHg). In this case, if the aim is to improve cerebral DO_2_, cerebral triggers should be preferred to target Hb levels in acute brain-injured patients, although this strategy may result in systemic hypoperfusion and non-cerebral organ dysfunction. Thus, decision to initiate RBCT in brain-injured patients remains a critical challenge for clinicians in the absence of specific monitoring tools.

Finally, future studies should also consider the use of EPO and its derivates to slowly increase Hb and minimize the risk of anemia and exposure to RBCT in such patients. Moreover, EPO may exert dose-dependent neuroprotective actions, including anti-inflammatory, anti-apoptotic, and endothelial effects, when administered in the early phase after injury, at least in experimental models [95, 96]. In the human setting, a small retrospective study showed some potential benefits for erythropoiesis-stimulating agents after TBI [97], while significant concerns about the increased risk of thrombotic events or even mortality have been raised in patients with stroke or polytrauma [98, 99]. In a recent trial, Robertson et al. [86] showed no significant benefits on neurological recovery of EPO compared with placebo in 200 severe TBI patients. In another randomized study, Nichol et al. [100] showed that EPO given once per week for a maximum of three doses (*n* = 308) did not reduce the proportion of patients with poor neurological outcome compared with placebo (*n* = 298, 44 versus 45 %, *p* = 0.90). Unfortunately, the RBCT requirement was similar between groups, although the amount of RBC packs transfused over the study period was not specifically reported in this study.

## Conclusions

Anemia is common among brain-injured patients and associated with worse outcome. RBC transfusions may rapidly increase Hb levels in such patients but are also associated with poor outcome and complications. Few clinical studies on the optimal transfusion strategy have been performed in this setting and are biased by significant confounders. A restrictive RBCT policy should be implemented in such patients, in particular if they are awake and conscious, unless poor tolerance to anemia (e.g. ischemic heart disease) are present. An individualized transfusion strategy is warranted in poor-grade patients, using different tools to detect global/cerebral hypoxia, although the reliability of such approach need to be adequately validated.

## Abbreviations

AJDO_2_: arterio-jugular oxygen
CaO_2_: arterial oxygen content
CBF: cerebral blood flow
CI: confidence interval
CMRO_2_: cerebral metabolic rate of oxygen
DO_2_: oxygen delivery
EPO: erythropoietin
Hb: hemoglobin
HIF: hypoxia-inducible factor
ICH: intracranial hemorrhage
ICU: intensive care unit
LPR: lactate to pyruvate ratio
NO: nitric oxide
OR: odds ratio
PbtO_2_: brain tissue oxygen pressure
RBC: red blood cells
RBCT: red blood cell transfusion
SAH: subarachnoid hemorrhage
ScvO_2_: vena cava oxygen saturation
StO_2_: brain oxygen saturation
SvjO_2_: venous saturation in the jugular vein
TBI: traumatic brain injury
